# Participant Retention in the Veterans Health Administration’s MOVE! Weight Management Program, 2010

**DOI:** 10.5888/pcd9.120056

**Published:** 2012-07-19

**Authors:** Sara M. Locatelli, Min-Woong Sohn, Bonnie Spring, Sattar Hadi, Frances M. Weaver

**Affiliations:** Author Affiliations: Min-Woong Sohn, Hines VA Hospital, Hines, Illinois, and Northwestern University, Chicago, Illinois; Bonnie Spring, Northwestern University, Chicago, Illinois; Sattar Hadi, Hines VA Hospital, Hines, Illinois; Frances M. Weaver, Hines VA Hospital, Hines, Illinois, and Loyola University, Maywood, Illinois.

## Abstract

**Introduction:**

Participant retention is a frequent concern in structured weight-management programs. Although research has explored participant characteristics influencing retention, little attention has been given to the influence of program characteristics. The objective of this study was to examine how program characteristics relate to participant retention in the Veterans Health Administration’s weight-management program, MOVE!

**Methods:**

We conducted semistructured interviews with coordinators of 12 MOVE! programs located throughout the United States, 5 with high participant retention rates and 7 with low rates. We transcribed and descriptively coded interviews and compared responses from high- and low-retention programs.

**Results:**

Characteristics related to retention were provider knowledge of and referral to the program, reputation of the program within the medical facility, the MOVE! meeting schedule, inclusion of physical activity in group meetings, and involvement of the MOVE! physician champion. MOVE! introductory sessions, frequency of group meetings, and meeting topics were not related to retention. Coordinators described efforts to improve retention, including participant contracts and team competitions. Coordinators at 5 high-retention facilities and 1 low-retention facility discussed efforts to improve retention.

**Conclusion:**

Coordinators identified important program characteristics that could guide improvements to retention in group-based weight-management programs. Training for providers is needed to assist with referral decisions, and program planners should consider incorporating physical activity in group meetings.

## Introduction

Weight management is a major health problem in the United States; more than two-thirds of Americans are overweight or obese (1) and, therefore, at increased risk for chronic health conditions, including diabetes, hypertension, and cardiovascular disease (2). Although people who attend structured weight-management programs achieve better weight loss than those who manage weight on their own (3), participant retention in weight-management programs is problematic (4). In clinical trials of obesity treatment programs, drop-out rates range from 30% to 50% (5-7); a large-scale study of the commercial Jenny Craig program found a drop-out rate of 58% (8).

Our study examined the Veterans Health Administration (VHA) weight management program, MOVE!, which was developed by the VHA National Center for Health Promotion and Disease Prevention (NCP). Most (98%) of the approximately 150 VHA medical centers across the country offer a MOVE! program (9). The program follows evidence-based obesity treatment guidelines and has a comprehensive, multidisciplinary approach to weight management (10). MOVE! shares many features of commercial weight-management programs, including self-management support via telephone or Internet, and group meetings involving discussion, activities, and short lessons (eg, reading food labels). Required MOVE! program staffing includes a program coordinator, a physician champion, and interdisciplinary staff. The coordinator supervises staff, sets local goals, ensures program compliance with national standards, and tracks program outcomes. The physician champion is responsible for patient safety, promoting outreach to other services in the VHA and the community, and providing medical consultations for MOVE! participants. Beyond staff requirements, however, facilities have great flexibility in how they organize program components, particularly the group meetings that are the focus of this study.

Previous research on MOVE! identified program characteristics (eg, leadership support, well-defined staff roles, use of support and maintenance sessions) associated with successful program implementation (9,11,12) and better patient weight-loss outcomes (13). Less research has addressed program retention. Participants who actively participate in supervised weight-management programs are more successful at losing weight than participants who fail to complete these programs (8). In 2010, 42% of MOVE! participants stopped attending group sessions after 1 visit (12).

Patient characteristics, such as age, depression, and dieting history, influence retention in weight-management programs (7,14,15). The influence of program characteristics, such as meeting topics and schedules, on participant retention in MOVE!, has not been fully explored. The purpose of our study was to examine differences in program characteristics between high- and low-retention MOVE! programs.

## Methods

### Participants and settings

We selected participants using dissimilar case sampling (16), in which cases are purposively selected to maximize differences for a variable of interest. NCP identified MOVE! programs with high and low retention, based on the average number of visits per patient enrolled in the group program. After stratifying by geographic location (urban vs rural), and facility complexity level (a category assigned by VHA to reflect levels of patient risk, teaching and/or research, and patient volume, and assessed as high, medium, or low) (17), we selected 20 programs, 10 with high participant retention and 10 with low retention. We determined through discussion with local MOVE! staff that coordinators would be the most knowledgeable about the group program; therefore, we contacted program coordinators (n = 20) via e-mail and invited them to participate in an interview about their program. Eleven coordinators (10 dietitians and 1 registered nurse) agreed to participate (response rate, 55.0%), representing 12 MOVE! programs. (One participant was coordinator at 2 facilities, both with low retention. Because the purpose of this was to explore program differences related to retention, and these 2 programs had key differences in implementation, these programs were analyzed separately.)

### Data collection

Our study was reviewed and approved by the authors’ local VHA institutional review board. Coordinators were informed that the purpose of the interview was to gather information about their MOVE! program to identify factors related to patient retention, and to discuss any efforts to improve retention at their facility. The first author (S.M.L.), an experienced qualitative researcher, conducted telephone interviews in 2010 using a semistructured interview guide (Appendix). Three members of the study team developed the guide; it was then reviewed for clarity and completeness by local MOVE! program staff. Interviews lasted approximately 30 minutes and were recorded and transcribed verbatim.

### Data analysis

Two research team members developed categories of program stages (referral, orientation, group meetings, and postprogram follow-up) based on earlier research describing patient assessment, referral, program attendance (individual, group, or intensive), and optional follow-up (18). Additionally, coordinators were asked to discuss several topics relevant to MOVE! overall; these responses were categorized as general MOVE! issues. We used descriptive coding techniques (19) to analyze content and used NVivo 8 qualitative data analysis software (QSR International, Doncaster, Victoria, Australia) to code responses in program stages. Members of the study team met throughout coding and analysis to discuss the codebook and the coding and categorization of coordinator statements. After coding, 3 members of the research team compared frequencies and responses between high- and low-retention programs to determine which characteristics differentiated them.

## Results

### Referral method

Referral method was not related to differences in retention (Table 1). In 10 programs (5 low- and 5 high-retention), patients were referred to MOVE! by a primary or specialty care provider; providers were reminded through a clinical alert to offer MOVE! to patients who had a body mass index (BMI) of 25 kg/m^2^ or higher and, if the patient was interested, request a MOVE! consultation. In 5 programs (3 low- and 2 high-retention), patients were also self-referred; self-referral was the primary referral method in 2 programs, both with low retention.

**Table 1:** Relationship of Participant Retention to VHA Facility Complexity, Location, Location Characteristics, Referral, and Orientation in 12 VHA MOVE! Weight Management Programs, 2010

VHA Facility	Complexity Category^a^	Local Environment	Geographic Region	MOVE! Visits per Patient^b^	MOVE! Retention Category^c^	Referral to MOVE!	Orientation Delivery Method
1	Low	Urban	South	1.0	Low	Provider, self	None
2	High	Rural	West	1.1	Low	Self	Individual
3	High	Urban	West	1.1	Low	Self	Group
4	High	Urban	Midwest	1.7	Low	Provider, self	Group
5	Medium	Rural	Northeast	2.0	Low	Provider	Group
6	High	Urban	Midwest	2.3	Low	Provider	None
7	High	Urban	South	3.8	Low	Provider	Group
8	High	Urban	West	5.5	High	Provider	Group
9	High	Urban	Midwest	6.1	High	Provider	Individual
10	Medium	Rural	Northeast	7.3	High	Provider, self	Individual
11	Medium	Rural	Northeast	10.3	High	Provider	Individual
12	Medium	Rural	South	10.6	High	Provider	Individual

Abbreviation: VHA, Veterans Health Administration

^a^ Category assigned by the Veterans Health Administration to reflect levels of patient risk, teaching or research, and patient volume.

^b^ Average number of visits per patient enrolled in the MOVE! group program.

^c^ Low-retention facilities were selected from the lowest retention facilities, as measured by mean number of visits per patient, stratifying by geographic location, local environment, and facility complexity; high-retention facilities were selected from the highest retention facilities in the same way.

Although referral method appeared unrelated to retention rate, provider support for and knowledge about the program differed between high- and low-retention programs. Coordinators at 4 low-retention programs said patients attended orientation without knowing why they were there or were often referred by providers who offered little discussion of MOVE!: “[The patients] don’t know they’ve been referred . . . they don’t know anything about it . . . I . . . think maybe the doctors just say, ‘Do you want to lose weight?’ and the patient says yes. . . . Maybe the doctors just don’t know what MOVE! is” (low-retention program). Only 1 coordinator of a high-retention program noted any such problems: “I would like to see a more consistent understanding among providers of what [MOVE!] is.”

Program reputation may also be related to retention. One coordinator from a high-retention program discussed a positive reputation: “Our program has a good reputation here. We . . . always have plenty of recruits, plenty of veterans referred to us.” On the other hand, a coordinator from a low-retention program discussed a negative reputation: “MOVE! has gotten a lot of negative attention in the past. . . . The doctors got bad feedback.”

### Orientation session

After referral, participants in 10 programs (5 low- and 5-high-retention) attended an orientation session. Five programs held orientation sessions individually (1 low- and 4 high-retention) and 5 as a group (4 low- and 1 high-retention). One facility that allowed walk-ins without referral at their orientation session had low retention. Overall, use of orientation sessions was unrelated to differences in retention.

After orientation, patients in all programs could select self-management support by telephone, Internet, or group meetings. Coordinators said veterans were encouraged to enroll in group meetings.

### Group meetings

Retention was unrelated to whether veterans could join mid-cycle (Table 2). Patients in 6 programs (3 low- and 3 high-retention) began attending classes immediately after orientation; in the remaining 6 programs (4 low- and 2 high-retention), patients waited until a new cycle began: “I have them wait until the beginning. . . . It’s easier to remember where they started . . . [and they develop] camaraderie with the other participants” (low-retention program).

**Table 2:** Meeting Frequency, Number, Planning Materials, Format, and Physical Activity, 12 Veterans Administration MOVE! Weight Management Programs, 2010

VHA Facility	Meeting Frequency	Length, h	No. of Sessions^a^	Planning Materials^b^	Presentation Format^c^	Physical Activity^d^
1	1 Meeting only	2	1	MOVE! handouts, professional knowledge^e^, veteran feedback^f^	Interactive	No
2	Weekly	1	12	MOVE! handouts, guest speaker	Didactic	Yes
3	Monthly	2	12	MOVE! handouts, guest speaker^g^	Didactic	No
4	Weekly	2	8	MOVE! handouts, professional knowledge^e^, veteran feedback^f^	Interactive	Yes
5	Weekly	2	4	MOVE! handouts, professional knowledge^e^, veteran feedback^f^	Interactive	No
6	Weekly	1	8	MOVE! handouts, veteran feedback^f^, guest speaker^g^	Didactic	No
7	Weekly	1	7	MOVE! handouts, professional knowledge^e^	Didactic	No
8	Weekly	1.5	9	MOVE! handouts, professional knowledge^e^, veteran feedback^f^	Didactic	Yes
9	Every other week	1	9	MOVE! sessions, other WM program, professional knowledge^e^	Didactic	Yes
10	Weekly	1	4	MOVE! handouts, professional knowledge^e^	Didactic	Yes
11	Weekly	1.5	8	MOVE! session, veteran feedback^f^	Didactic	Yes
12	Weekly	1.5	12	MOVE! handouts, other WM program, professional knowledge^e^	Interactive	No

Abbreviation: VHA, Veterans Health Administration; WM, weight management.

^a^ Number of group sessions per class cycle.

^b^ Materials used to plan group program sessions.

^c^ Method in which group session information is presented. Didactic involves lecture for presentation of materials; interactive focuses on group discussion and activities.

^d^ Whether program staff involved participants in physical activity as part of MOVE! group sessions.

^e^ Knowledge of the coordinator or any MOVE! interdisciplinary staff.

^f^ Any participant feedback, whether gathered formally (eg, program evaluations) or informally (eg, 1-on-1 discussions).

^g^ Any person not considered part of the MOVE! program who leads group sessions.

Program retention also was unrelated to frequency of meetings; 5 low- and 5 high-retention programs offered classes once per week or every other week. The number of classes offered in a cycle also did not relate to retention; 9 programs (5 low- and 4 high-retention) offered 6 or more sessions in a cycle. On the other hand, retention rate differed by flexible scheduling; 2 high-retention programs offered more than 1 meeting time, to accommodate different schedules, whereas no low-retention programs did so.

When planning the program and determining which sessions to hold, all coordinators used handouts and activities from the MOVE! website; only 2 coordinators, both of high-retention programs, used complete lesson plans provided by NCP. Most coordinators instead supplemented MOVE! materials with presentations by guest speakers, the coordinators’ professional knowledge, and materials from other weight-management programs.

All programs offered sessions on diet and nutrition and psychology; for 11 programs, diet and nutrition topics made up more than half of program content. Eight programs (6 low- and 2 high-retention) used lecture primarily; 4 programs (1 low- and 3 high-retention) were interactive, using group discussion and activities. Whereas 9 programs discussed physical activity in class, high-retention programs were more likely to incorporate physical activities into class sessions; 2 low- and 4 high-retention programs included physical activities, such as walking, chair exercises, and balance/stretching in class.

All coordinators identified retention as a major issue in the group program. Availability of resources, such as space, was a barrier for both high- and low-retention programs: “Our classroom right now, if I would have everyone show up . . . it would be extremely crowded. I kind of bank on some people not showing up” (high-retention program). “[The veterans] don’t like where we are. It’s a small area. . . . There’s nowhere else. . . . They haven’t allotted us space” (low-retention program).

Another barrier to improving retention was patient mix. Coordinators of 2 high- and 5 low-retention programs noted poorer attendance and participation among women: “I notice that some women don’t like to be in a room with 12 men. That makes them uncomfortable” (high-retention program). “The women have different issues than the men. . . . They would probably feel more comfortable with a group of their own” (low-retention program).

Two programs (1 low- and 1 high-retention) created a separate MOVE! program for women through collaboration with the Women’s Health Service: “We didn’t have a lot of women participating in the group classes, and when we did, their attendance was sporadic. . . . [Participants] really enjoy just having a women’s-only class and not having to deal with any pressures [with discussing certain issues] in front of a man” (low-retention program).

Despite resource limitations, coordinators discussed strategies for improving retention. Coordinators from high-retention programs were more likely to discuss active efforts to improve retention (5 high- vs 1 low-retention), including participant contracts addressing active participation and attendance and team competitions and challenges. Coordinators of low-retention programs, on the other hand, often discussed poor retention as inevitable: “I know that my retention rate is going to drop off by about 60% to 80% after the first week.” “I tell [patients] in their first meeting . . . ‘Look around, because half of you won’t make it to class 3.’ They just don’t come.”

### Postprogram follow-up

At the end of the program cycle, participants were presented with follow-up options. One option that all programs allowed is re-attending classes. Re-attendance was subtly discouraged in 3 programs (2 low- and 1 high-retention) because of space constraints or observations of poor engagement by returning participants: “After they’ve been through [MOVE!] a time or 2, it’s like a social event for them. They come and visit and they’re not very active with trying to lose weight” (high-retention program).

Ten programs (5 low- and 5 high-retention) had a support group for past participants. Support groups met weekly or monthly; topics were determined by what was timely (eg, dieting during the holidays) or of interest to participants. Four programs (2 low- and 2 high-retention) also used TeleMOVE! for follow-up. TeleMOVE!, a new home-based program, uses a telephone-connected device to collect and transmit information between the participant and their VHA facility (11): “Some of them, now that we have that program, are choosing to follow up that way. It’s a little more convenient for them, especially if they have a long travel distance” (high-retention program).

### General MOVE! issues

Some general infrastructure issues (program location, staff/leadership support) were related to program retention. Eight programs (3 low- and 5 high-retention) were located in the facility’s Nutrition and Food Services; 4 (all low-retention) were located in the facility’s Primary Care department. Additionally, 7 programs (5 low- and 2 high-retention) lacked a physician champion or had one in name only. Only 2 low-retention programs had a physician champion who was involved in teaching classes. Although 2 high-retention programs also lacked a physician champion, coordinators at these facilities felt they had strong support from other sources, such as facility leadership: “Our primary care service line manager is very supportive. He’s all gung-ho for [MOVE!]” (high-retention program). “[Leadership has] been supportive of innovation. . . . I’ve had a lot of autonomy to manage the program” (high-retention program).

Although the job description specified the skills and attributes of the physician champion, coordinators felt the role of the physician champion was unclear, which was a barrier to greater involvement. Coordinators reported they had sufficient staff members to conduct classes but lacked personnel for other MOVE! activities, such as scheduling patients, informing facility staff about the program, and planning sessions: “I’m a one-man band here. I do all of the scheduling, all the canceling . . . the support groups, and there’s getting to be too many patients” (high-retention program). “[I would like] all those things that [MOVE!] is supposed to have . . . it’s supposed to be a team effort. . . . It just doesn’t happen. . . . I would like it to be the way they advertise it — a team” (low-retention program).

When coordinators were asked what patient characteristics they felt influenced retention, they listed age, health status, and personal investment or motivation. Driving distance was identified as a factor by coordinators of 6 programs (2 low- and 4 high-retention). These coordinators dealt with driving distance in 1 of 2 ways. One way, used by 2 high- and 2 low-retention programs, was to enroll patients in TeleMOVE!. Another method, used by 2 high- and 1 low-retention programs, was to offer MOVE! at affiliated community-based outpatient clinics (CBOCs): “[Our medical center is] not very centrally located . . . and so depending on . . . how far they have to travel, that’s probably the biggest [issue]. . . . We have the CBOCs involved, so they can travel to a CBOC and see the same thing” (high-retention program).

## Discussion

Factors that may relate to MOVE! participant retention were provider knowledge and referrals, program reputation, inclusion of physical activity, engagement of physician champions, and multiple meeting times. Although our research design precludes us from inferring that these factors caused retention, many of these factors have been identified as important for other MOVE! outcomes (9,11-13) suggesting that efforts to target these factors may improve multiple program performance metrics.

Providers and participants may be more supportive of the program if they had a better understanding of purpose and content. The MOVE! physician champion could take on a more active role to promote change through social influence, knowledge sharing, and interventions (20). Woolf (21) recommends targeted interventions, including formal education, to address lack of knowledge, and systems to encourage and reinforce behaviors, such as incentives, clinician reminder systems, and feedback on patient outcomes. Although clinician reminder systems exist in many facilities, no facility in this study used other reinforcement systems. Feedback on patient outcomes could further engage providers by demonstrating the effectiveness of the program. Motivational interviewing (22), a collaborative approach to behavior change, is one method providers can use to encourage patients to make diet and physical activity changes.

Because physical activity during meetings was related to high retention in this study, program planners should consider adding these activities. More weight loss occurs when weight-management programs include physical activity rather than nutrition only (23). MOVE! participants are likely to vary in age, physical ability, and fitness, so programs should accommodate these differences to maximize participant involvement. Coordinators who included physical activity frequently used stretching, chair exercises, and similar activities, which are easy to implement and can be completed by participants at multiple ability levels.

Regardless of retention, all programs discussed diet and nutrition and health behavior; there were no differences in materials used for planning classes. Ability to self-refer or join the meetings mid-cycle were also unrelated to retention, indicating that improving access to the program may not necessarily increase retention. Finally, although coordinators perceived resource issues, few programs had a dedicated budget, and coordinators of high- and low-retention programs were equally likely to discuss staff and space constraints.

Because little is known about weight-management program features that influence retention, the qualitative approach of this study was appropriate. Our findings provide a foundation for improving retention, albeit one that needs to be confirmed by further research. The study has limitations. We did not randomly select VHA facilities; instead, facilities were selected to represent 2 levels of retention, high and low, to highlight differences between programs. We did not interview program staff other than coordinators. Finally, coding procedures and codebook definitions were discussed with the research team, and the framework used to categorize responses was based on prior research. However, we did not conduct a full inspection of coding reliability.

Obesity has become a significant public health concern, receiving growing attention from VHA and the medical community. The results of this study can help support the MOVE! program and non-VHA weight-management programs in determining best practices. Though past research has focused largely on participant characteristics related to retention, program characteristics may also influence retention.

## Appendix. Semistructured Interview Questions

1. Describe for me how veterans are referred to and join the MOVE! group program.
  - If not addressed: can they join at any time during the cycle of meetings?
  - If not: what do they do in the meantime?
2. How frequently do you hold group meetings?
  - Prompts: What day of the week? Length?
3. When planning the group program, how do you decide which sessions to hold?
  - What materials do you use to plan the sessions?
  - Do you use the same sessions every cycle?
4. Tell me about a typical group meeting. About how many people attend, on average?
  - What do you do at the meetings?
  - Prompts: physical activity, mindfulness-based approaches, low-sodium diet, alcohol consumption, DASH diet
5. At the end of a cycle, when does the next cycle begin?
6. Are there limits to how many cycles a participant can complete?
7. What outcomes do you measure?
  - Prompts: weight loss, improved cardiovascular risk, better diet, increased activity
  - Do you make special efforts to recruit special populations (wheelchair, blind)? If not, are there barriers to including these groups in MOVE!?
8. What kind of support are you receiving from your facility leadership?
  - Prompts: resources, doctors refer patients, voluntary service support, etc.
9. What Veterans Affairs staff are involved in the program? Do they have dedicated/protected time? Do they volunteer?
10. What do you think is the retention rate in your program?
  - Are you making any organized efforts to improve retention?
11. What patient characteristics do you think influence whether someone stays in the group program?
12. What characteristics of your program or facility do you think influence retention?
13. If you could change anything about the MOVE! group program, what would it be?
